# Improved awareness of physical activities is associated with a gain of fitness and a stable body weight in breast cancer patients during the first year of antineoplastic therapy: the BEGYN-1 study

**DOI:** 10.3389/fonc.2023.1198157

**Published:** 2023-08-11

**Authors:** Cosima Zemlin, Julia Theresa Schleicher, Laura Altmayer, Caroline Stuhlert, Carolin Wörmann, Marina Lang, Laura-Sophie Scherer, Ida Clara Thul, Lisanne Sophie Spenner, Jana Alisa Simon, Alina Wind, Elisabeth Kaiser, Regine Weber, Sybelle Goedicke-Fritz, Gudrun Wagenpfeil, Michael Zemlin, Georges Steffgen, Erich-Franz Solomayer, Carolin Müller

**Affiliations:** ^1^ Department of Gynecology, Obstetrics, and Reproductive Medicine, Saarland University Medical Center, Homburg, Saar, Germany; ^2^ Department of General Pediatrics and Neonatology, Saarland University Medical Center, Homburg, Saar, Germany; ^3^ Institute for Medical Biometry, Epidemiology and Medical Informatics (IMBEI), Saarland University Campus Homburg, Homburg, Saar, Germany; ^4^ Department of Behavioural and Cognitive Sciences, Institute for Health and Behaviour, University of Luxembourg, Esch-sur-Alzette, Luxembourg; ^5^ Department of Outcomes Research, Anesthesiology Institute, Cleveland Clinic, Cleveland, OH, United States

**Keywords:** breast cancer, physical activity, resting heart rate, fitness tracker, MET, body composition, weight, COVID - 19

## Abstract

**Background:**

Breast cancer is the most frequent cancer in women. Reduced physical activity and overweight are associated with poor prognosis. Breast cancer patients have a high risk to gain weight, lose muscle mass and reduce physical activity during therapy. Concepts are urgently needed to motivate patients to engage in physical activity.

**Methods:**

110 non-metastatic breast cancer patients were included in the prospective observational BEGYN-1 study. Physiological parameters and body composition were measured before the start of therapy and then quarterly for one year. Patients used a fitness tracker and documented their physical activity in a diary throughout the study.

**Results:**

Although the patients were not offered any guided exercise, and despite the restrictions during the COVID-19 pandemic, they increased their physical activity (metabolic equivalent of task (MET) -minutes): p<0.001), physical fitness (decreasing resting heart rate: p=0.001) and did not gain weight (median - 0.4kg) over the course of the study.

**Conclusion:**

Improved awareness of physical activity is associated with an increase in physical activity, fitness, and a stable weight during the first year of therapy in breast cancer patients. Counselling at diagnosis should motivate patients to engage in physical activity, wear a fitness tracker and document activities.

## 1 Introduction

The diagnosis of breast cancer often represents a traumatic event in patients that can result in reduced physical activity, weight gain and deteriorating physical fitness (1, 2). Especially weight gain during chemotherapy has already been recognized to be a problem in breast cancer patients (3). Weight gain, as well as reduction of physical activity and fitness are known to negatively influence the success of cancer therapy and overall outcome (4–7). In addition, obesity is a risk factor for the development of breast cancer in the first place. Especially occurrence of hormone-receptor positive breast cancer in postmenopausal women could be associated with obesity (8).

Physical activity is playing an important role in primary prevention of breast cancer, as physical activity is associated with a reduction of primary breast cancer and recurrent breast cancer (9). Based on a large population-based study among breast cancer patients, physical activity before and after chemotherapy was associated with significantly reduced hazard ratios of recurrence and mortality (10). However, in this study, physical activity was assessed using questionnaires (10). To better understand the consequences of physical activity during anticancer therapy on side effects, body composition and physiological parameters, we performed the prospective BEGYN-1 study (11, 12).

Van Gemert et al. showed that 5.5% of postmenopausal breast cancer cases are attributable to physical inactivity and 8.8% to overweight, making this together an even greater risk than smoking (4.6%) and alcohol (6.6%) (13). Healthcare professionals should counsel patients in ways that increase awareness of the importance of physical activity as well as a healthy lifestyle (14). But not only breast cancer patients can benefit from physical activities. The importance of physical activity and the positive influence on the quality of life in patients with other cancer entities, e.g., lung and colorectal cancer, has been shown previously (15, 16).

There is an urgent need to develop and implement novel strategies that motivate newly diagnosed breast cancer patients to engage in physical activity from diagnosis throughout the entire course of anticancer therapy. Several previous studies used an interventional approach to measure the influence of strength and/or endurance sports (7, 17–20). A meta-analysis found that supervised interventions regarding physical activity might be a greater independent motivator compared than partly supervised or unsupervised interventions (21). Supervised exercise showed a stronger effect on primary outcomes, quality of life, and physical function than the type or amount of recommended exercise (21). Recent systematic reviews demonstrated, that in combination with diet, exercise intervention led to improved cardiorespiratory fitness, muscular strength, body composition, quality of life, fatigue, anxiety, depression, and sleep compared to control groups (22–24). Furthermore, in long-term studies, healthy bodyweight resulting from regular physical exercise was associated with a better quality of life compared to overweight or obese women who gained body weight after diagnosis (25).

However, maintaining the level of physical activity is difficult after the completion of a short-term exercise intervention (26). It is important to provide patients with sustainable motivational concepts after an intervention (27). Activity diaries alone might offer some motivation, but there are considerable discrepancies between this form of self-assessment and other methods to quantify physical activity (28).

The use of fitness trackers has been recommended for a more objective assessment (29, 30). Fitness trackers have been successfully used to quantify physical activity by tracking step counts of patients undergoing oncological treatment (31). Furthermore, fitness trackers continuously measure the heart rate and determine the resting heart rate. The resting heart rate is an important marker not only for physical fitness but correlates with longevity and with the risk of all-cause mortality in breast cancer patients (32, 33). A systematic review showed that the use of a fitness tracker in combination with motivational interviews had the most consistent positive effect on adherence to physical activity (34). During the COVID-19 pandemic, an increased use of fitness trackers was observed that was positively associated with physical activity (35). However, multiple studies showed that physical activity decreased, and body weight increased during the Covid-19 pandemic in German and US-American population (36–38).The aim of the BEGYN-1 study was to assess the physical activity, resting heart rate and body weight, as well as body composition during the first year of newly diagnosed nonmetastatic breast cancer patients. We aimed to detect differences between the first and the last three months during the first year after breast cancer diagnosis, thus after completion of most acute oncologic therapies (e.g., chemotherapy, radiotherapy, surgery). According to the national guideline for breast cancer, moderate physical activity was recommended to all patients (39, 40). Physical activity was measured by using a fitness tracker as well as a personal activity diary. Quarterly routine follow-up visits were used for monitoring physiological parameters and body composition and for evaluation of physical activity.

## 2 Patients and methods

### 2.1 Patients

Patients were enrolled in the BEGYN-1 study between September 2019 and January 2021 (11). The baseline study visit was scheduled before the start of any antineoplastic therapy or intervention (e.g., surgery, chemotherapy) but after information by a medical doctor about the diagnosis. The BEGYN-1 study lasted one year in total, and all patients presented themselves every three months for their follow-up visits. Clinical assessment was obtained at each visit, including anamnesis, measuring body weight, as well as blood pressure, resting heart rate, and bioelectrical impedance analysis. Therefore, nutritional and hydration deprivation (except water) was required prior to the measurements and patients presented themselves in sportswear.

**Figure 1:**
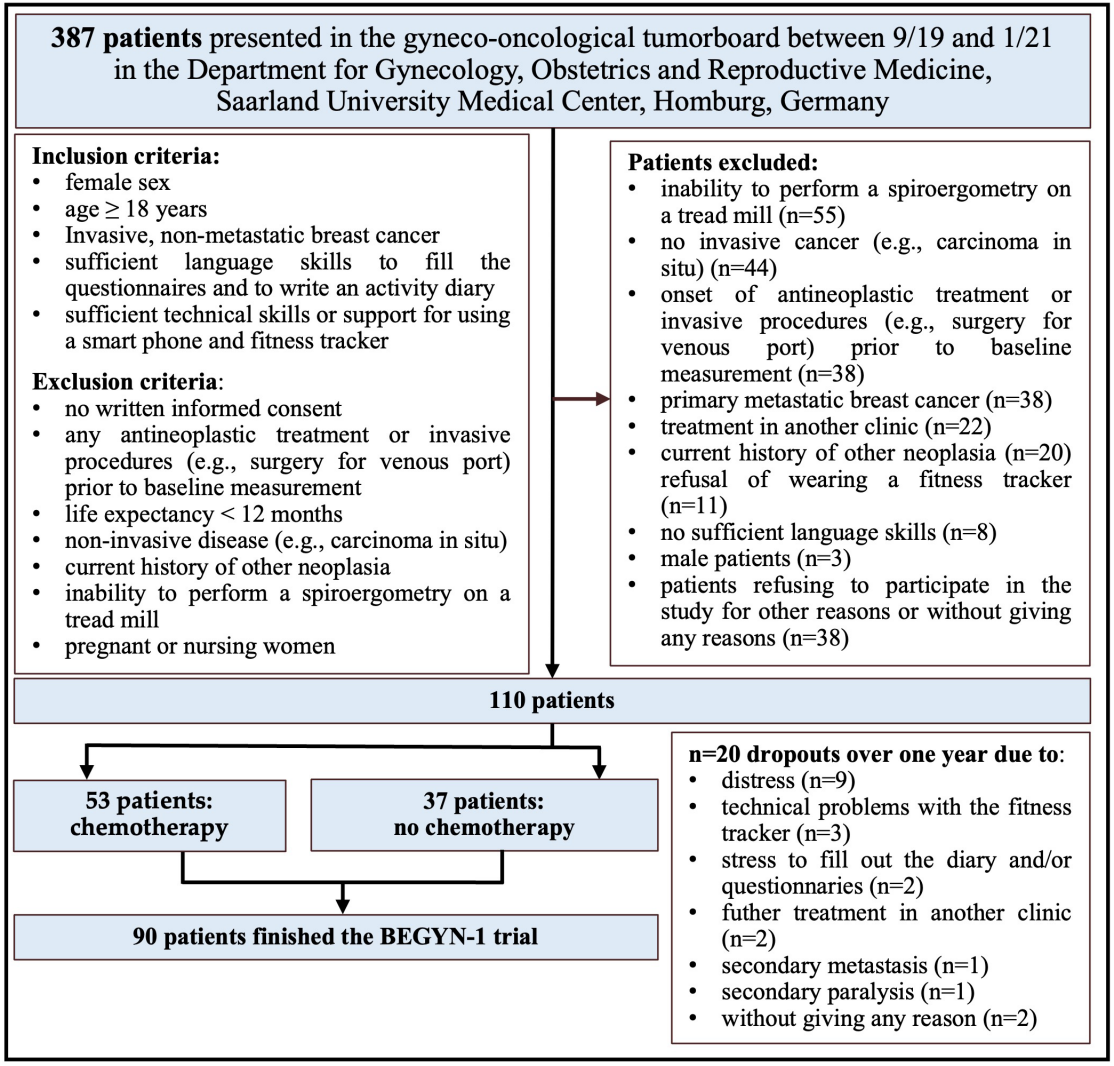
Recruitment and flow of participants in the BEGYN-1 trial.

The study was approved by the ethics committee of the Medical Association of Saarland (study # 229/18). Inclusion and exclusion criteria are presented in **Figure 1**. All patients provided written consent. Documentation of clinical characteristics (e.g., age, BMI, Karnofsky performance status scale), as well as histopathological parameters was performed.

### 2.2 Methods

All patients received their own fitness tracker (Fitbit charge 3™, Fitbit Inc., San Francisco) at the baseline visit. The fitness tracker was linked to a smartphone and patients were motivated to wear the fitness tracker during the whole day, ideally at any time (including nights) to measure steps, physical activity, and heart rate (29). Weekly fitness reports of the fitness tracker were read out during the follow-up visits.

Additionally, all patients documented their sporting activities and calorie consumption in their individual study diary. The diary included daily notes on the type and length of physical activities. The patients were asked to transfer the measurement results from the fitness tracker to the diary. They could add comments in a free text field. The diary was evaluated during the follow-up visits by study staff together with the patients.

METs (Metabolic Equivalents of Task) can be used to compare different activities regarding their energy consumption. One MET corresponds to the conversion of 3.15 ml oxygen per kilogram of body weight per minute in women. In the current study, METs were calculated using information from the fitness tracker, as well as the study diary. METs were calculated for all days throughout the year and mean values were calculated for individual days, weeks, and months. MET minutes (MET-min) were obtained by multiplying MET activity with duration of the activity (MET-min*_activity_* = MET*_activity_* × duration*_activity_*) (41, 42). When “MET-min” is mentioned in the following manuscript, this term refers to MET-min*_activity_*. The energy expenditure (kcal) and the duration (minutes) of certain activities were documented from the fitness tracker’s actigraphy. Body weight (kg) was measured weekly by the patients themselves. A distinction was made between total activity and workout. The daily calorie consumption, measured by the fitness tracker, was defined as the total activity. Periods of rest, or periods where the fitness tracker was not worn (e.g., nights) were included and the Fitbit app calculated the basal metabolic rate for these periods.

In the case of missing data imputation was carried out according to previously published methods by Stephens et al. (43). Missing values for the total activity occurred when the tracker was not worn or only partially worn during the day or when no value could be determined due to technical problems and/or lack of cooperation on the part of the patients. Where possible, the missing values were imputed according to standard procedures (43). Briefly, the requirement was that at least 50% of the data on daily calorie consumption was available in the relevant diary. At least three (if possible five) plausible references were used, and the arithmetic mean of the reference values was used as the estimated value for the missing value. Days with the same activities (e.g., walking and swimming or no sport) or with the same general condition (e.g., nausea or hospitalization) as on the day with the missing value were selected as references. To impute the activities as completely as possible, missing recordings were supplemented according to the following algorithm: If energy expenditure was not reported for an activity, an estimate was calculated based on other recorded exercises of the same type and intensity (at least three, if possible five references). For this purpose, an arithmetically averaged quotient was calculated with the duration or the route of the references. Implausible outlier values measured by the fitness tracker (e.g., an apparent extremely long walking distance), that were in conflict with the activity diary, were replaced by the patient’s self-assessment in consultation with the patient, so that the energy consumption was estimated based on the patient information according to the above scheme. Consideration of both, fitness tracker measurements and activity diary thus lead to the most complete assessment of physical activity (43). In accordance with the published recommendations, we differentiated between data that was missing at random or systematically, e.g., when the patient only wore the fitness tracker during sporting activities but not during daily life (43).

Body composition was assessed using bioelectrical impedance analysis. For the bioelectrical impedance analysis (BIA) (TANITA 601 scale™, Tanita Europe BV, Stuttgart), the patients stood barefoot on a body scale and holding sensors in both hands. Based on a dual frequency bioelectrical impedance analysis at four measuring points, the scale yields information about the muscle, fat, bone, and water content of the whole body as well as individual compartments (44, 45).

### 2.3 Statistics

Statistical analyses were performed using SPSS as IBM Corp. Released 2021. IBM SPSS Statistics for Windows, Version 28.0. Armonk, NY: IBM Corp. Qualitative parameters (e.g., tumor stage) are presented as frequencies and quantitative parameters are given as mean with standard deviation or as median with range. Normal distribution was assessed by Shapiro-Wilk testing. Inter-group comparison was performed using the Mann-Whitney-U test. Assessments over time were compared with the Wilcoxon signed-rank test and repeated measures analysis of variance (Friedman test) combined with pair-wise comparisons adjusted for multiple testing according to the Bonferroni method. Correlations were analyzed using Spearman Rank correlation. Thus, ΔMET-min/week (ΔMET-min/week = MET-min/week [week 40-52] minus MET-min/week [week 1-13]) were used to correlate physical activity with weight, visceral fat, body fat, muscle mass and BMI between the first three months and the last three months within the first year after diagnosis.

## 3 Results

In total, 110 patients were included in the BEGYN-1 trial (**Figure 1**). In the period between September 2019 and January 2021, n=387 patients with high suspicion or newly diagnosed breast cancer were presented to the tumor board at the breast center of the Department of Gynecology, Obstetrics and Reproductive Medicine, Saarland University Medical Center, Homburg/Saar, Germany. Of these n=387 patients, n=181 met the inclusion criteria (**Figure 1**). N=110 patients signed the declaration of consent after being informed by a physician. N=20 patients dropped out of the study (**Figure 1**). The patients were 26 to 81 years old when included in the study.

Ninety patients completed the study until the final one year follow up assessment and were used for the analysis. Of these n=90 patients, n=53 patients received chemotherapy (CHT). N=37 patients did not receive chemotherapy (NCHT). Median length of chemotherapy was 147 days (min. 76, max. 189 days). All patients received surgical treatment and n=75 patients (83%) underwent radiotherapy. Median length of radiotherapy was 37 days (min. 18, max. 69 days). All patients received surgical treatment and n=72 patients (80%) endocrine therapy. Patients’ characteristics regarding tumor entity, tumor subtype, and tumor stage, as well as age are listed in **Table 1**. Timing of oncological treatment throughout the year is illustrated in **Table 2**.

**Table:** Table 1 Patient characteristics including tumor entity (histology, grading), tumor stage (TNM-classification) and age in all patients/chemotherapy (CHT)/no chemotherapy (NCHT).

		CHT (n=53)	NCHT (n=37)	All patients (n=90)
**Tumor entity**	NST	44 (83.0%)	31 (83.8%)	75 (83.3%)
	invasive lobular	7 (13.2%)	2 (2.7%)	9 (10.0%)
	others	2 (3.8%)	4 (10.8%)	6 (6.7%)
**cT**	T0*	2 (3.8%)	1 (2.7%)	3 (3.3%)
	T1	29 (54.7%)	34 (91.8%)	63 (70.0%)
	T2	19 (35.8%)	2 (5.4%)	21 (23.3%)
	T3	1 (1.9%)	0 (0.0%)	1 (1.1%)
	T4	2 (3.8%)	0 (0.0%)	2 (2.2%)
**cN**	N0	38 (71.7%)	35 (94.6%)	73 (81.1%)
	N+	15 (28.3%)	2 (5.4%)	17 (18.9%)
**cM**	M0	53 (100%)	37 (100%)	90 (100%)
Grading**	G1	0 (0.0%)	10 (27.0%)	10 (11.1%)
	G2	19 (35.8%)	23 (62.2%)	42 (46.7%)
	G3	34 (64.2%)	3 (8.1%)	37 (41.1%)
**Age**	26-30 years	2 (3.8%)	1 (2.7%)	3 (3.3%)
	31-40 years	10 (18.9%)	3 (8.1%)	13 (14.4%)
	41-50 years	13 (24.5%)	9 (24.3%)	22 (24.4%)
	51-60 years	15 (28.3%)	10 (27.0%)	25 (27.8%)
	61-70 years	11 (20.8%)	10 (27.0%)	21 (23.3%)
	71-78 years	2 (3.8%)	4 (10.8%)	6 (6.7%)

*3 patients had a recurrent tumor in the lymph nodes without tumor manifestation in the breast, thus T0**Grading not available in one NCHT patient.

**Table 2:** Treatment (chemotherapy, radiotherapy, and surgery) according to time quartiles.

Therapy	0-3 months	3-6 months	6-9 months	9-12 months	Total therapy
**Chemotherapy**	53 (100%)	52 (98%)	15 (29%)	2 (4%)	53 (100%)
**Surgery**	55 (61%)	12 (13%)	22 (25%)	1 (1%)	90 (100%)
**Radiotherapy**	27 (36.5%)	7 (9.5%)	37 (50%)	3 (4%)	74 (100%)
**Endocrine therapy**	37 (41%)	46 (51%)	65 (72%)	72 (100%)	72 (100%)

All in all, n=53 patients underwent chemotherapy, all (n=90) patients had surgery, n=74 patients received radiotherapy, and n=72 patients received endocrine therapy. Values are given as n= number of patients and percentage (%).

**Figure 2:**
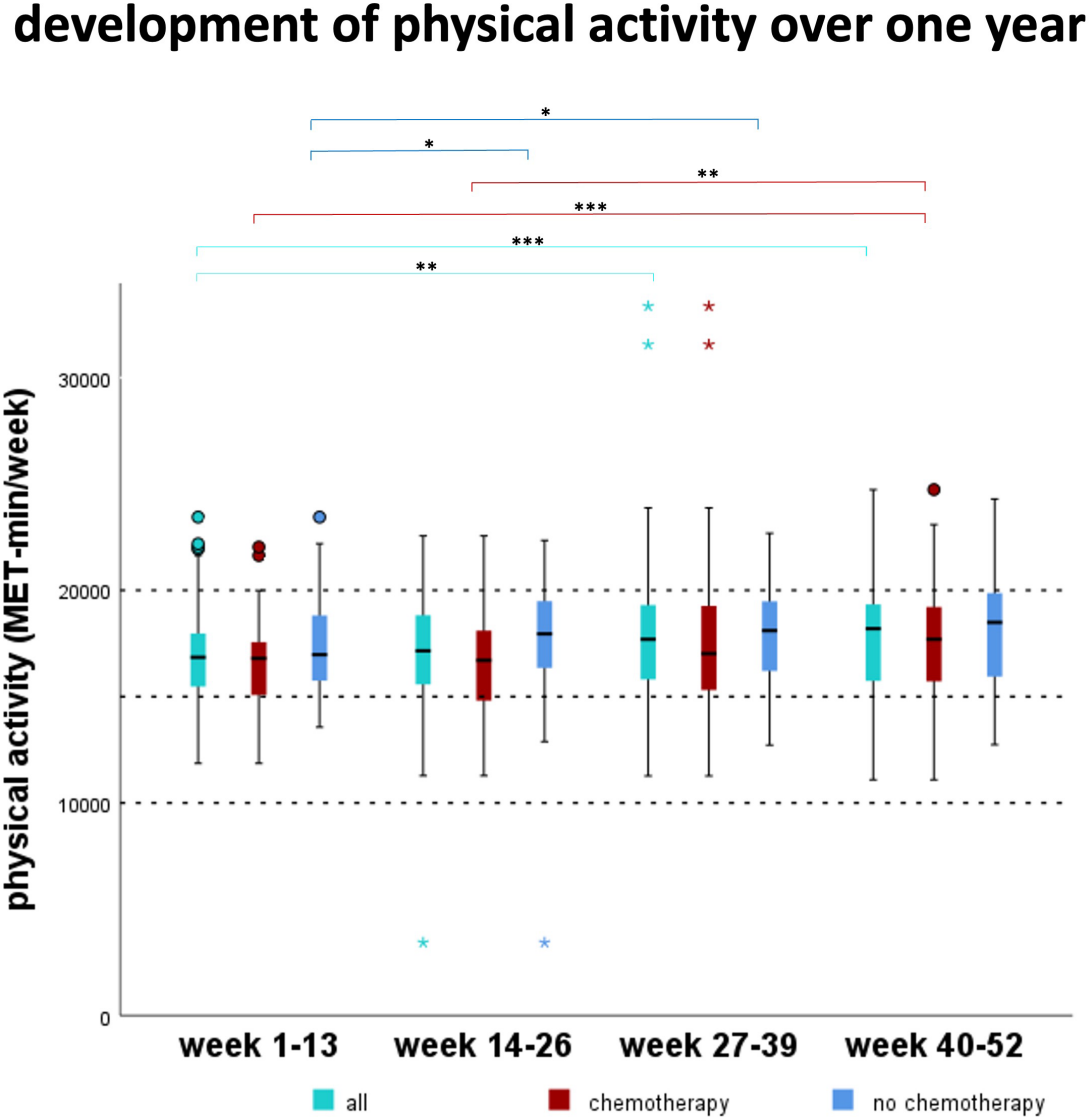
Development of the physical activity over one year. MET (Metabolic equivalents of task). Over all patients, the MET-mins significantly increased between week 1-13 compared to weeks 27-39 and 40-52 (p<0.01). In patients receiving chemotherapy, MET increased significantly between weeks 1-13 and 14-26 compared to week 40-52 (p<0.01). In patients who did not receive chemotherapy, MET increased significantly between week 1-13 compared to weeks 14-26 and 27-39 (p<0.05). *p<0.05, **p<0.01, ***p<0.001.

Overall, physical activity increased significantly from 16845 MET-min/week (median) in the first quarter of the study to 18114 MET-min/week in the last quarter of the study (p<0.001) (**Figure 2**). In the two subgroups, physical activity increased from 16804 MET-min/week to 17697 MET-min/week (CHT, p<0.001) and from 16973 MET-min/week to 18357 MET-min/week (NCHT, p=0.07), respectively. Furthermore, the ranges of activity increased over time in both therapy groups: 11860 to 22037 MET-min/week (CHT) and 13564 to 23443 MET-min/week (NCHT) in the first quarter of the study to 11093 to 24736 MET-min/week (CHT) and 12738 to 24300 MET-min/week (NCHT) in the last quarter of the study. There was no significant difference related to physical activity between the two therapy groups (**Table 3**).

**Table 3:** Physical activity and body composition in all patients/chemotherapy (CHT)/no chemotherapy (NCHT) at two different time points (baseline visit/end of trial) and difference between baseline visit and end of the study (Δ).

	Median first visit	Range first visit	Median deviation after one year	First versus last visit*	CHT versus NCHT Δfinal-first visit*
MET-min/week					
All (n=90)	16845	11860 – 23443	+995	p < 0.001	
CHT (n=53) NCHT (n=37)	16804 16973	11860 – 22037 13564 – 23443	+1209 +709	p < 0.001 p = 0.07	p = 0.396
Resting heart rate [1/min]					
All (n=90)	77	58 - 138	-5	p < 0.001	
CHT (n=53) NCHT (n=37)	78 74	58 – 138 62 - 114	-5 -5	p < 0.001 p = 0.037	p = 0.625
Bodyweight [kg]					
All (n=90)	69.4	45.6 – 107.4	-0.4	p = 0.259	
CHT (n=53) NCHT (n=37)	70.2 69.3	51.0 – 107.4 45.6 – 92.8	-0.9 +0.6	p = 0.145 p = 0.850	p = 0.650
Visceral fat [absolute]					
All (n=90)	7	2 - 14	0	p = 0.509	
CHT (n=53) NCHT (n=37)	7 7	2 – 14 2 – 13	0 0	p = 0.943 p = 0.325	p = 0.281
Body fat [%]					
All (n=90)	34.8	16.9 – 48.5	+0.1	p = 0.936	
CHT (n=53) NCHT (n=37)	34.9 33.4	16.9 – 48.5 16.9 – 44.4	-0.7 +0.6	p = 0.247 p = 0.165	p = 0.800
Muscle mass [kg]					
All (n=90)	43.6	34.4 – 55.8	-0.2	p = 0.180	
CHT (n=53) NCHT (n=37)	44.4 42.3	34.4 – 55.8 35.2 – 51.2	-0.1 -0.6	p = 0.479 p = 0.216	p = 0.628
BMI [kg/m²]					
All (n=90)	26	19 – 39	0	p = 0.250	
CHT (n=53) NCHT (n=37)	26 26	19 – 39 19 – 35	0 0	p = 0.148 p = 0.898	p = 0.256

MET, Metabolic equivalents of task. BMI, body mass index. *2-sided Mann Whitney-U test.

**Figure 3:**
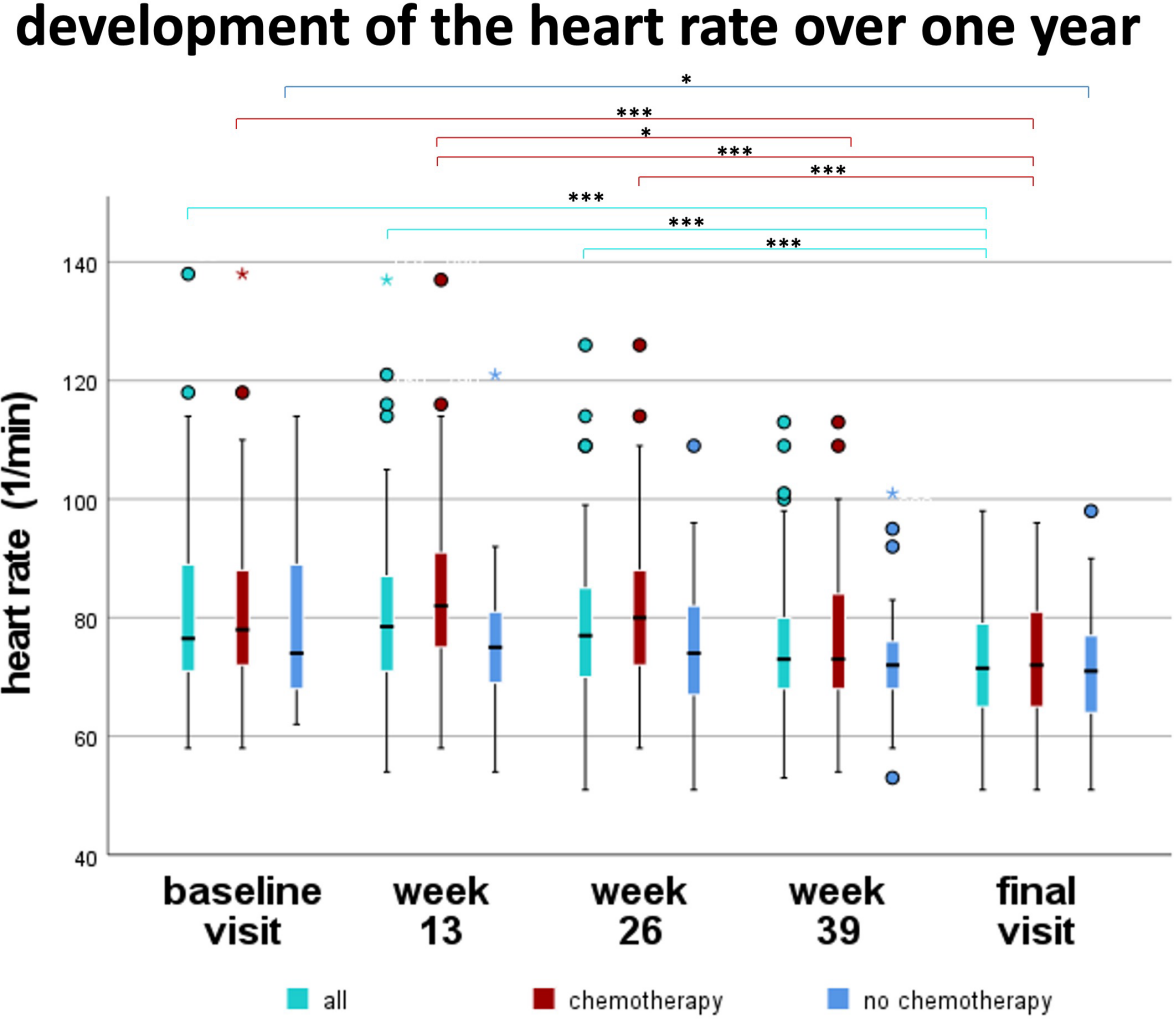
Development of the resting heart rate over one year. Over all patients, heart rate decreased significantly between baseline visit, week 13, and week 26 compared to final visit (p<0.001). In patients receiving chemotherapy, heart rate increased from week 1 to week 13. Afterwards, heart rate decreased significantly in chemotherapy patients comparing week 13 to 39 (p<0.05) and week 13 to final visit (p<0.001). *p<0.05, **p<0.01, ***p<0.001.

The resting heart rate of all patients decreased significantly during the study period from 77/min at the baseline visit to 72/min at the final visit at the end of the study period (p<0.001) (**Figure 3**, **Table 3**). More precisely, the patients who received chemotherapy (CHT) as well as those who did not receive chemotherapy (NCHT) therefore also showed a significant reduction of the heart rate during the investigation period from 78/min to 72/min (median, CHT, p<0.001) and 74/min to 71/min (median, NCHT, p=0.037), respectively. Regarding the patients who received chemotherapy an additional increase in heart rate occurred from first week to week 13. This represents an increase in heart rate during chemotherapy (**Figure 3**).

**Figure 4:**
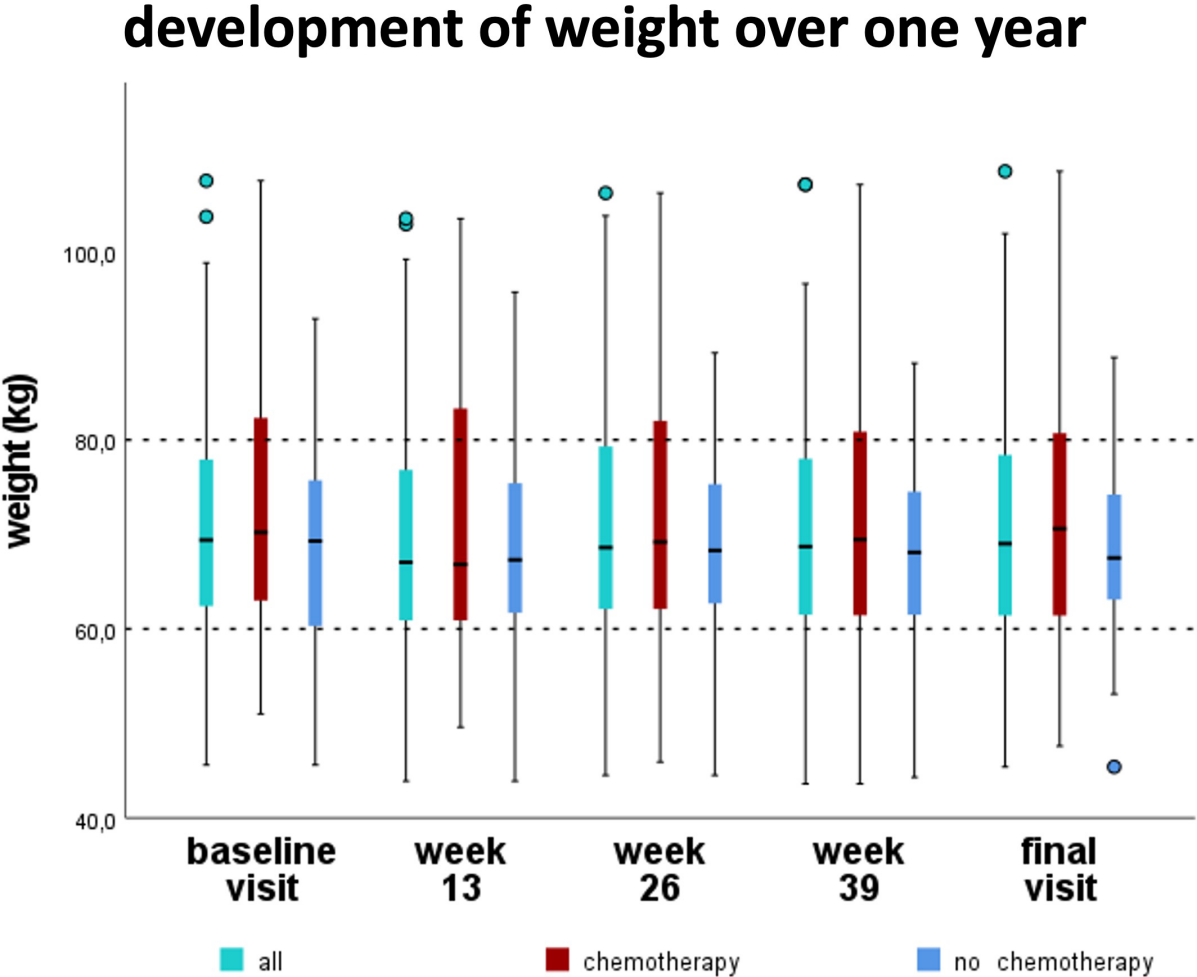
Development of the bodyweight over one year. Median body weight slightly decreased from 69.4kg (baseline visit) to 69.0kg (final visit) during the first year in newly diagnosed breast cancer patients, without reaching statistical significance.

The average weight of all patients decreased not significantly by 0.4 kg (p = 0.259) during the study period (**Figure 4**, **Table 3**). The weight changes of the patients receiving chemotherapy (CHT) and of those who did not receive chemotherapy (NCHT) also showed no significant differences over time (p = 0.145/p = 0.850) nor between CHT and NCHT (p = 0.650). CHT- patients had no significant loss of weight (median -0.9kg), body fat (median -0.7kg), muscle mass (median -0,1kg) and reduction of their BMI (median -0.2kg/m²) (**Table 3**). There was no significant difference between the NCHT and CHT groups for the body composition (**Table 3**).

To test the hypothesis that the quantity of physical activity might influence physiological parameters, we studied the correlation between the physiological parameters with the deviation of physical activity during the study period.

**Figure 5:**
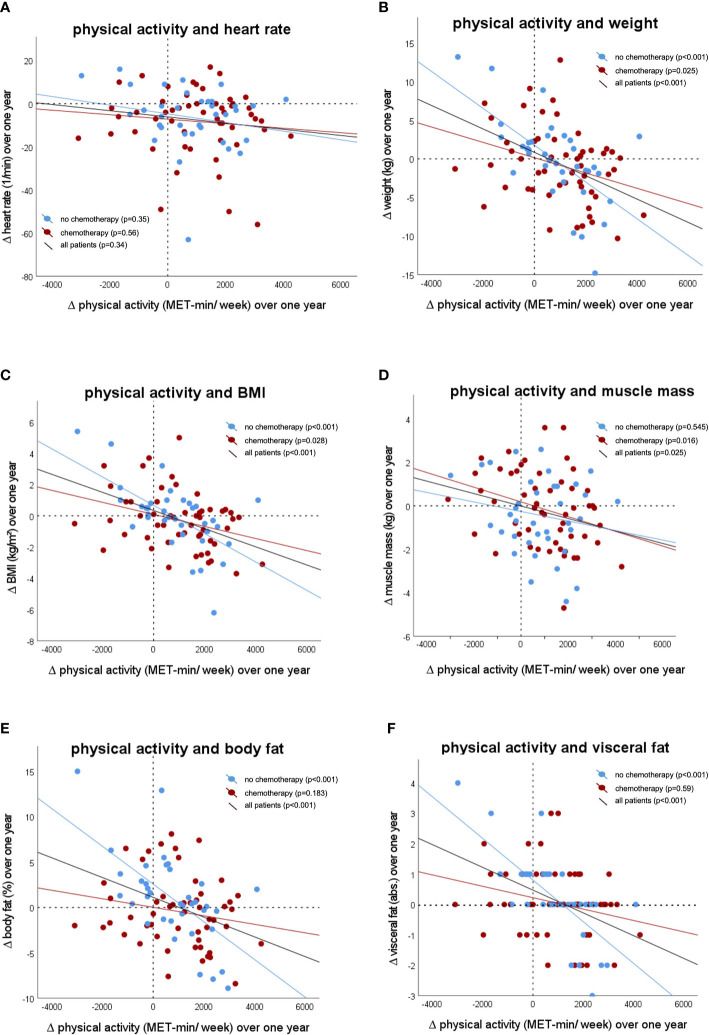
Correlation between the change of physical activity during the study period with **(A)** resting heart rate (correlation coefficient: -0.1; p=0.34), **(B)** body weight (correlation coefficient: -0.4; p<0.001), **(C)** Body mass index (BMI) (correlation coefficient: -0.4, p<0.001), **(D)** muscle mass (correlation coefficient: -0.2; p=0.03), **(E)** body fat (correlation coefficient: -0.4; p=0.001), **(F)** visceral fat (correlation coefficient: -0.5; p<0.001).

Over the course of the study year, a significant correlation for the entire patient collective concerning weight, visceral fat, body fat, muscle mass and BMI was observed comparing week 1-13 to week 40-52. Correlating the examined variables with the ΔMET-min/week (ΔMET-min/week = MET-min/week [week 40-52] minus MET-min/week [week 1-13]) over the course of the study year yielded a significant correlation for the entire patient collective for weight, visceral fat, body fat, muscle mass and BMI (**Figure 5**). The heart rate did not correlate significantly with the ΔMET-min/week, neither when considering all patients nor when examining the different therapy groups. The muscle mass of the NCHT patients, as well as the body fat and the visceral fat of the CHT patients, did not correlate significantly with the ΔMET-min/week over the course of the study year.

## 4 Discussion

The BEGYN-1 study revealed that the use of a fitness tracker and an activity diary are associated with an increase in physical activity and physical fitness as well as a stable body weight in breast cancer patients after the first year of therapy. Moreover, we observed a negative correlation between the extent of physical activity and changes of body weight, BMI, body fat, and visceral fat over time.

The influence of physical activity during anticancer therapy on fitness, remains poorly understood (10). Thus, the aim of this study was to assess the physical activity, fitness, physiological parameters, and body composition in newly diagnosed non-metastatic breast cancer patients during the first year of treatment. Since other studies reported a decrease in physical activity and fitness and an increase in weight gain during anticancer therapy (1, 2), routine counselling appears to be insufficient to achieve a long-lasting effect on the patient’s motivation. Guided successful short-term interventions were either not assessed with a long-term follow up (46) or they had little long-term effect on physical activity (26). Thus, we sought to identify factors that might motivate the patients to improve their lifestyle over the long term.

The BEGYN-1 study was designed as an observational study. All patients received a fitness tracker, were supposed to write an activity diary and were undergoing quarterly fitness assessments. While in intervention studies it is usually unclear how much the participants exercise outside of the interventions, the BEGYN study starts right here and aims to fully record physical activity throughout all days of the year. Although, patients did not receive guided exercise, we found evidence that the study participants were highly motivated to improve their lifestyle. Notably, 60% of the eligible patients agreed to participate in the BEGYN-1 study. This indicates that the majority of the newly diagnosed breast cancer patients were interested in actively addressing their physical condition even during the earliest stage of their disease and prior to the initiation of anticancer treatment. In agreement with this finding, the positive short-term and long-term effect of motivational interviews had been previously shown (34). We hypothesize that addressing the positive effects of physical activity during the initial disclosure of the diagnosis was perceived by the patients as a possibility to take control over the course of the disease and improve the feeling of self-efficiency. This has been shown to have a long-lasting positive effect on the psychological coping with the disease and to correlate with an improved outcome (47). The effect of higher self-efficiency and autonomy in cancer patients undergoing counseling for physical activity has been previously shown in different cancer entities (48).

In our cohort, a higher increase of weekly MET-min during the study period was associated with an improved physical status: We found a beneficial correlation between the extent of physical activity, bodyweight, BMI, and body fat mass. Thus, the BEGYN-1 study patients experienced similar benefits over a one-year period as interventional studies with supervised exercise (7, 17–20). Importantly, Zhou et al. have shown that a three-month wearable-based lifestyle intervention may help reduce weight and improve body composition in breast cancer survivors (20). Similar to our findings, Zhou et al. reported a decrease in overall muscle mass in patients with elevated physical activity. This apparent paradox might be due to suboptimal protein intake during antineoplastic therapy, due to the predominance of endurance sports overweight training- especially in patients that underwent surgery- and/or due to a loss of postural muscles as part of the weight loss (20).

Recommendation of physical activity is already included in national and international guidelines (7, 39, 40, 49). However, optimal intensity and duration of physical activity is not clear (49). Thus, according to the guidelines (7, 39, 40), patients were advised to engage in moderate physical activity, but no upper limit was mentioned. Even though, none of the BEGYN-1 study patients showed any sign of overexertion or other negative side effects of too much physical activity, further research is needed to individualize recommendations for the level of physical activity to aim for (50, 51). The increased physical activity and fitness achieved in the BEGYN-1 study might have multiple other consequences that are known to be associated with increased physical activity in breast cancer patients, e.g., increased bone health (52), reduced cancer related fatigue (53), and reduced cancer related cognitive impairment (54).

Strengths of our study are the high number of patients, the inclusion prior to therapy induction, the dual assessment of physical activities by using a fitness tracker and a diary, and the long follow up period of one year. METs are as a standardized quantification of physical activity, a validated method to summarize and compare differing physical activities (e.g., Nordic walking, swimming, cycling and weight training) (41, 42). This design allows to identify long-lasting effects on health parameters and minimizes potential transient confounders such as effects of perioperative immobility. Furthermore, improved physical activity was seen despite the Covid-19 pandemic. Previous studies revealed a reduction of physical activity during Covid-19 pandemic and especially during lockdowns (55). The biggest part of the BEGYN-1 study was carried out during Covid-19 pandemic, so patients were not able to participate in group sport or go to the gym, as most group activities were not allowed during Covid-19 pandemic.

The study results must be interpreted with caution due to some limitations: Due to ethical concerns, we did not include a control group that was deprived of counselling according to the guidelines (7, 39, 40). Thus, we can only hypothesize that the improvement of physical activity and fitness and the stability of body weight was a consequence of the motivation that was caused by participating in the study. Medical guidance, such as motivational interviews, using an activity diary or wearing a fitness tracker might contribute to additional motivation since patients knew, that corresponding data was evaluated. However, this hypothesis is strongly supported by other studies (20). Furthermore, physiological parameter (like heart rate) might be influenced due to higher level of function in some patients. These individuals might be more capable in participating in physical activity. Moreover, bioimpedance measurements and the use of fitness trackers are readily available, but they also have technical limitations (20) and missing values had to be calculated to approximate the total MET-mins (41, 42).

## 5 Conclusion

We conclude that prevention of decreased physical activity and fitness, as well as gain of weight during the first year after diagnosis of early breast cancer, even during the Covid-19 pandemic, is possible. Improved awareness of physical activity could lead to an increase in physical activity, fitness, and a stable body weight during the first year after diagnosis of early breast cancer. Patients should be encouraged to exercise, wear a fitness tracker, and/or document activities. By positively influencing their lifestyle, patients get back their self-control, increase their quality of life and long-term outcome.

## Data availability statement

The datasets generated during the current study are available from the corresponding author on reasonable request.

## Ethics statement

The studies involving humans were approved by ethics committee of the Medical Association of Saarland (study # 229/18). The studies were conducted in accordance with the local legislation and institutional requirements. The participants provided their written informed consent to participate in this study.

## Author contributions

CZ wrote the study conception and design. CZ implemented the study in the clinic, recruited the patients and supervised the study. E-FS and MZ gave advice for the study design and supervised the study. Material preparation and data collection were performed by JTS, LA, CS, CW, ML, L-SS, IT, LS, JAS, AW, EK, RW, and SG-F. The data analysis was performed by CZ and GW. The first draft of the manuscript was written by CM and CZ. All authors commented on previous versions of the manuscript. All authors contributed to the article and approved the submitted version.
